# Cytoarchitecture and myeloarchitecture of the sheep auditory cortex

**DOI:** 10.1111/joa.70072

**Published:** 2025-11-12

**Authors:** Camille Pluchot, Mélody Morisse, Maryse Meurisse, Jean‐Marie Graïc, Elodie Chaillou, Scott A. Love

**Affiliations:** ^1^ INRAE, CNRS, Université de Tours, PRC Nouzilly France; ^2^ UMR 1253, Imaging Brain & Neuropsychiatry iBraiN, Université de Tours, Inserm Tours France; ^3^ Department of Comparative Biomedicine and Food Science University of Padova Legnaro Italy

**Keywords:** auditory cortex, interneurons, myelin basic protein, ovine, parvalbumin, posterior ectosylvian gyrus

## Abstract

The auditory cortex is central to auditory perception, but its detailed structural organization in sheep (*Ovis aries*) has not been thoroughly investigated. In this study, we sought to address this gap by providing an in‐depth anatomical description of the cytoarchitecture and myeloarchitecture of the sheep auditory cortex, using cresyl violet staining and the neurochemical markers myelin basic protein and parvalbumin. Cresyl violet tissue samples from four sheep were used to characterize cortical layers and cellular composition, revealing a six‐layered organization with variations in cell density and distribution. Myelin basic protein staining highlighted myelinated regions, while parvalbumin staining identified the distribution of a subpopulation of GABAergic interneurons, indicating the potential location of the primary auditory cortex. Overall, the organization of the ovine auditory cortex aligns with findings in other mammals, suggesting a conserved neural architecture across species and supporting the idea of evolutionary conservation in auditory processing mechanisms.

## Graphical abstract

This article describes the cyto‐ and myeloarchitecture of the sheep auditory cortex. It outlines a six‐layered cortical organization, with variations in cellular types and densities. The potential location of the primary auditory cortex is indicated by parvalbumin immunostaining.

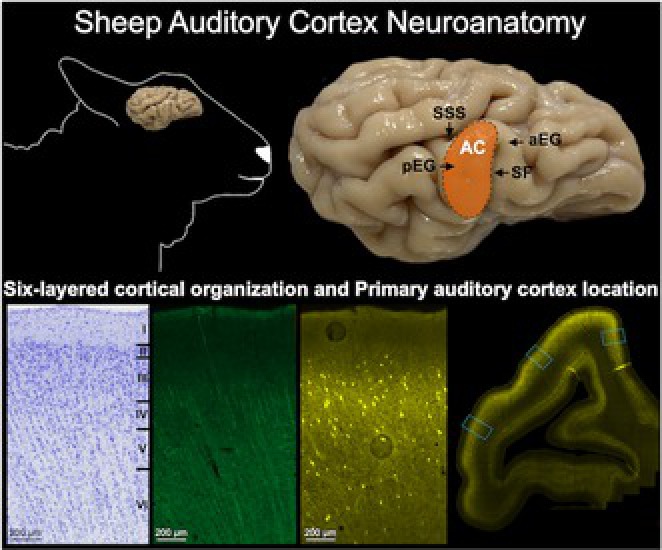

## 1 INTRODUCTION

As a gregarious prey species, sheep rely on multiple sensory modalities to communicate and monitor their environment. Understanding how they perceive and interact with their surroundings is fundamental to elucidating their cognitive capacities and emotional states, and to addressing key concerns in farm animal welfare. The sheep motor and somatosensory cortices have been extensively studied (Bagley, 1922; Dinopoulos et al., 1985; Gierthmuehlen et al., 2014; Johnson et al., 1974; King, 1911; Peruffo et al., 2019; Simpson & King, 1911; Woolsey & Fairman, 1946), and the primary visual cortex has received some attention (Clarke et al., 1976; Clarke & Whitteridge, 1976; Ebinger, 1975; Karamanlidis et al., 1979). Despite the central role of audition in social behavior, the neural mechanisms underlying auditory perception in sheep remain poorly characterized. Sheep have, however, been employed as a model for hearing loss through studies of auditory brainstem responses in the fetal and perinatal phases (Cook et al., 1987; Griffiths et al., 1994; Pierson et al., 1994, 1995), or middle ear histology (Roberto et al., 1989). However, the cortical regions of the auditory pathway remain largely unexplored.

To date, only two studies have investigated the sheep auditory cortex. In the first study, Michaloudi et al. (1986) injected a horseradish peroxidase retrograde tracer into several areas of the posterior ectosylvian gyrus, in the parietal area of Rose (1942). When the tracer was injected into two sites of the anterior part of the posterior ectosylvian gyrus, labeled cells were found exclusively in the ventral division of the medial geniculate nucleus (MGNv), which was known to be the final subcortical relay of auditory information before the primary auditory cortex in other mammals (Kaas, 2011; Oliver & Hall, 1978; Winer et al., 1977). Based on this specific connection, Michaloudi et al. (1986) concluded that the anterior part of the posterior ectosylvian gyrus corresponded to the sheep primary auditory cortex. Thus, it can be argued that this region contains the putative homologue of Brodmann area 41 and that the connectivity profiles of surrounding injection sites represent homologues of Brodmann areas 42 and 22 (Gupta, 2017). Decades later, while developing a high channel count neural recording system, Sahasrabuddhe et al. (2021) confirmed the auditory function of this region by recording cortical surface local field potentials in response to auditory stimuli. Together, these findings suggest that the sheep auditory cortex is located in parieto–temporal brain areas homologous to those of other mammals (Kaas, 2011).

Histological techniques, such as immunohistochemistry and neural tracing, have provided valuable insights into the cytoarchitecture and myeloarchitecture of the auditory cortex across various mammalian species (e.g., human: Hackett et al., 2001; non‐human primate: Hackett et al., 2001; mouse: Anderson et al., 2009; rat: Smith et al., 2012; guinea pig: Wallace et al., 2000; cat: Winer & Prieto, 2001; rabbit: De Venecia et al., 1998; ferret: Bajo et al., 2007; gleaning bat: del Martin Campo et al., 2014; African wild dog: Chengetanai et al., 2020). These studies consistently show that the auditory cortex is organized into six distinct cortical layers, with variations in thickness, cellular composition and distribution (i.e., pyramidal neurons, interneurons, and glial cells, depending on the cortical layers). In sheep, this six‐layered organization has been identified in the primary visual cortex (Graïc et al., 2022). However, the cytoarchitecture of the sheep auditory cortex remains unexplored.

The present study aims to provide a detailed description of the cytoarchitecture and myeloarchitecture of the sheep auditory cortex, using the localization established by the two aforementioned studies (Michaloudi et al., 1986; Sahasrabuddhe et al., 2021). We employed cresyl violet (‘Nissl’) staining to investigate the cytoarchitecture, and define the number and thickness of cortical layers, as well as the composition and distribution of cells within each layer. Additionally, we assessed the distribution of myelin basic protein (MBP), a marker of myelinated fibers, to investigate the cortical myeloarchitecture (Jeffrey et al., 1990). We also investigated the neurochemical properties of the sheep auditory cortex by quantifying the distribution of parvalbumin (PV), a marker of a subpopulation of GABAergic interneurons (Hof et al., 1999), which has been used to delineate the primary auditory cortex in various species (mice: Cruikshank et al., 2001; rabbits: McMullen et al., 1994; gerbils: Budinger et al., 2000; cats: Wallace et al., 1991; monkeys: Jones et al., 1995; Kaas & Hackett, 2000; Kosaki et al., 1997). Together, these approaches provide valuable insights into the structural organization of the sheep auditory cortex.

## 2 MATERIALS AND METHODS

### 2.1 Tissue sampling

**FIGURE 1:**
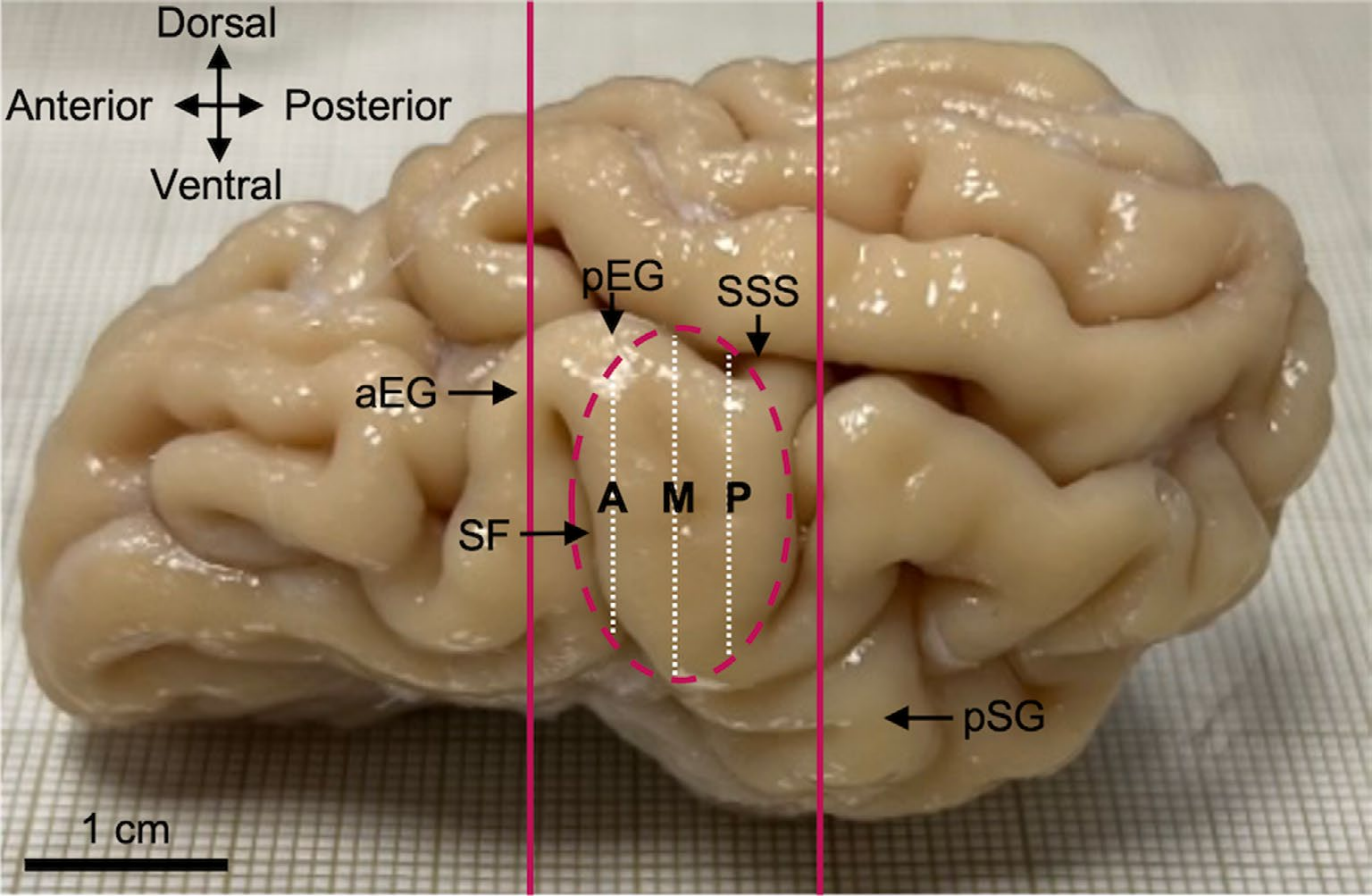
Identification of the auditory cortex (dashed red circle) within the tissue block delimited by the two vertical red lines. Lateral view of the left hemisphere of a sheep brain. For analysis, three sections (anterior (A), medial (M), and posterior (P)) were selected from each hemisphere to represent the antero‐posterior extent of the auditory cortex. Consistency across animals was ensured by selecting the sections based on the same anatomical landmarks. Sylvian Fissure (SF), anterior Ectosylvian Gyrus (aEG), posterior Ectosylvian Gyrus (pEG), Suprasylvian Sulcus (SSS) and posterior Sylvian Gyrus (pSG); grid paper: 1 cm × 1 cm.

This research was conducted in compliance with French and European guidelines for the housing and care of animals used for scientific purposes (European Union Directive 2010/63/EU). In accordance with the 3R principles of animal research, we chose to acquire the necessary biological tissue from animals that were reared and euthanized, independently of the present study; no experimental procedures were conducted on any live animals. The brains of four adult Ile‐de‐France sheep (3 ewes: 13507, 13403, 13155; and 1 ram: 03133), between 2 and 3 years of age, were collected at a local slaughterhouse (UEPAO, https://doi.org/10.15454/1.5573896321728955E12; agreement number G37‐175‐2). Sheep are generally considered to have a lifespan of up to 12 years. They typically reach puberty between 4 and 8 months of age and are regarded as physiologically mature/adult between one and one and a half years of age. The sheep were administered an intravenous injection of ketamine (5 mL). After confirming the loss of consciousness (i.e., absence of pupillary and palpebral reflexes), they were slaughtered by a licensed butcher. Then, the heads were immediately perfused in the carotid arteries with sodium nitrite at 37°C (2 L per sheep, 1% in sodium chlorure solution 0.9%), followed by 4% paraformaldehyde at 4°C (4 L per sheep, in phosphate‐buffered saline (PBS), 0.1 M, pH 7.4). The brain was extracted from the skull and post‐fixed with 4% paraformaldehyde for 24–48 h. It was washed in several PBS baths to remove excess paraformaldehyde, and immersed in a 20% sucrose cryoprotectant solution in PBS for at least 24 h to prevent the formation of ice crystals during subsequent freezing. Each brain was cut in half sagittally, through the interhemispheric fissure. Then, each hemisphere was sectioned into a coronal orientation tissue block of approximately 1.5 cm. The blocks were frozen with dry ice (−70°C) on the microtome stage, then cut into 40 μm thick coronal sections using a frozen microtome (Thermo Fisher Scientific, Sliding Microtome Microm HM 430, Germany). The coronal sections were stored at 4°C in PBS containing 0.1% sodium azide, until the respective staining protocols were performed. Every 20th section, corresponding to a distance of 800 μm, was mounted and stained with cresyl violet (see below) to visualize the cortical anatomy. These sections included the anterior part of the posterior ectosylvian gyrus, which has been identified as the auditory cortex in sheep (Michaloudi et al., 1986) and other mammal species (Kaas, 2011; Oliver & Hall, 1978; Winer et al., 1977). The anatomical boundaries of the anterior part of the posterior ectosylvian gyrus were defined as follows: anteriorly by the sylvian fissure and the anterior ectosylvian gyrus; posteriorly by the suprasylvian sulcus (SSS) and the medial part of the posterior ectosylvian gyrus; ventrally by the sylvian fissure; and dorsally by the SSS (Figure 1). Three sections per hemisphere covering the antero‐posterior extent of this region were selected for further analysis and are referred to as anterior, medial, and posterior. Anatomical landmarks of the cortical anatomy were used to consistently select these sections across individuals. Therefore, a total of 24 (4 animals × 2 hemispheres × 3 sections) cresyl violet, 12 MBP and 12 PV (2 animals × 2 hemispheres × 3 sections) adjacent sections were selected for analysis.

### 2.2 Cresyl violet staining

For each tissue block, one out of 20 sections (800 μm intervals) was mounted on double‐frosted gelatin glass slides (RS France, Beauvais) and stained with cresyl violet (Nissl protocol) to visualize the cortical anatomy along the anterior–posterior axis of the sheep auditory cortex. First, sections were immersed in a 0.5% cresyl violet solution for 15–20 min and then rinsed twice with tap water to remove excess dye. The sections were then dehydrated in three successive baths of 95% ethanol for 2 to 7 min and three successive baths in 100% ethanol for 2–7 min, depending on the number of sections in the slide staining rack. Finally, they were immersed in three successive baths of toluene for at least 5 min, coverslipped with Depex, stored at room temperature, and imaged 48 h later.

### 2.3 Immunohistochemistry

For immunohistochemistry the sections adjacent to the anterior, medial, and posterior sections analyzed in the cresyl violet protocol were mounted using the same procedure as described above. Sections were then permeabilized in PBS–Triton–Azide–BSA 1% (PBSTA–BSA) for 1 h at room temperature. Then, they were incubated in the primary antibodies solution (monoclonal mouse anti‐PV or monoclonal rat anti‐MBP in PBSTA–BSA 1%) for 48 h at 37°C (Table 1). Then, sections were rinsed in four successive baths of PBS for 10 min. They were incubated for 3 h at 4°C with the secondary antibodies solution, containing donkey anti‐mouse‐CY3 or donkey anti‐rat‐CY3 in PBSTA–BSA 0.1% (Table 1). Then, sections were rinsed in four successive baths of PBS for 10 min. The sections were mounted on slides and oven‐dried at 37°C for at least 1 day. This time was chosen to ensure that the sections were completely dry to prevent slide movement during imaging. Slides were coverslipped under Fluoromount‐G® (Southern Biotechnology, Birmingham, AL), protected from light, stored at 4°C, and imaged 48 h later.

**TABLE 1:** Primary and secondary antibodies used for the immunohistochemistry process.

	Antibody	Clonality/host	Manufacturing details	Dilution
Primary	Mouse anti‐PV	Monoclonal/Mouse	Swant Cat# 235 RRID:AB_10000343	1:5000
	Rat anti‐MBP	Monoclonal/Rat	Millipore Cat# MAB386 RRID:AB_94975	1:1000
Secondary	Donkey anti mouse‐CY3	Polyclonal/Donkey	Jackson ImmunoResearch Labs Cat# 715‐165‐151 RRID:AB_2315777 Minimal cross‐reaction with rat	1:1500
	Donkey anti rat‐CY3	Polyclonal/Donkey	Jackson ImmunoResearch Labs Cat# 712‐165‐153 RRID:AB_2340667 Minimal cross‐reaction with mouse	1:1500

### 2.4 Microscopic imaging and section analysis

#### 2.4.1 Cresyl violet staining: Brightfield acquisition

**FIGURE 2:**
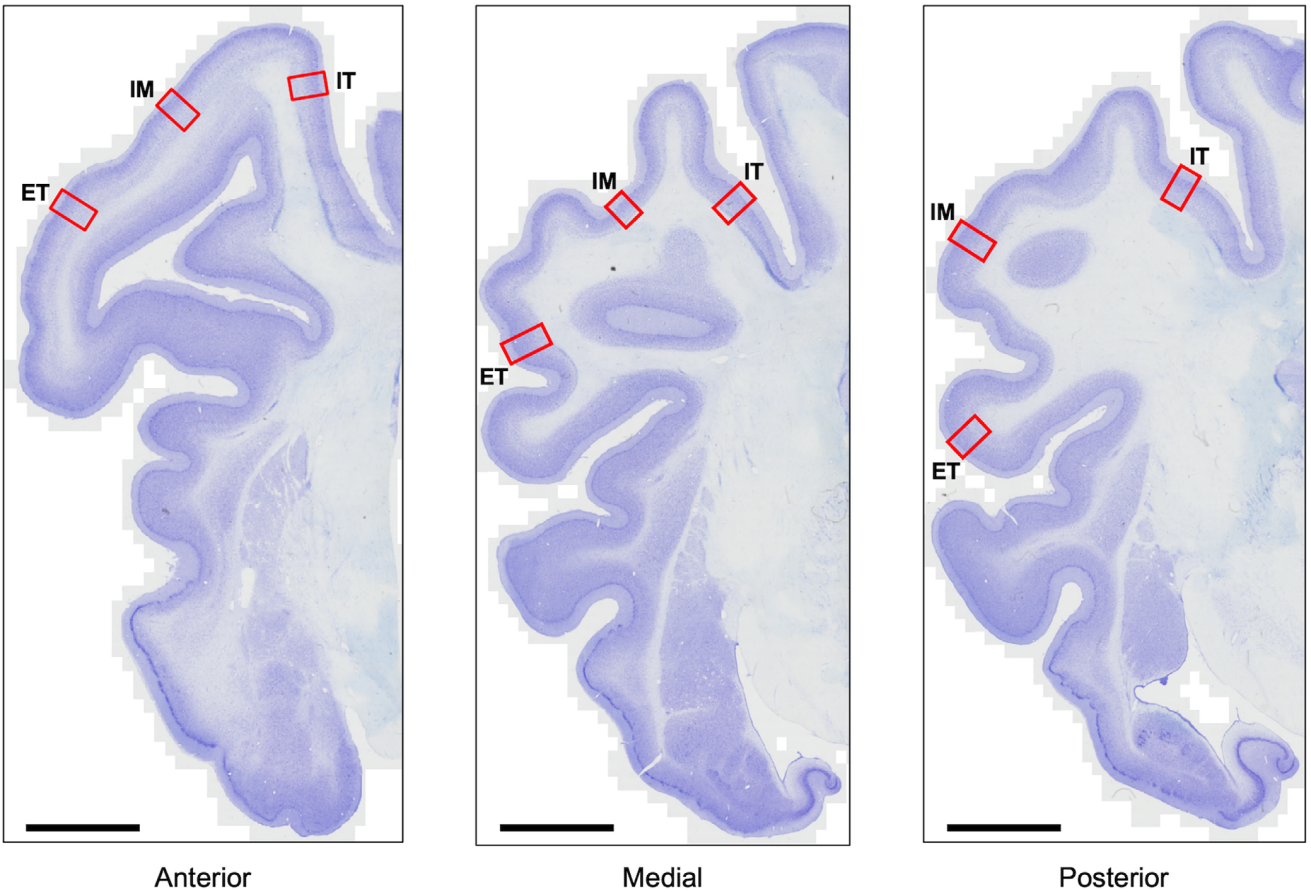
Positioning of ROIs on the sheep auditory cortex sections. Internal (IT), intermediate (IM), and external (ET) ROIs were positioned on relatively straight parts of the cortical surface of the anterior, medial, and posterior sections of the auditory cortex block. Images presented are from the left hemisphere of animal 03133. Scale bars: 5000 μm.

The 24 cresyl violet sections selected for further analysis were imaged using a digital slide scanner Axio Scan.Z1 (ZEISS®, Germany, with a 10× objective, in brightfield microscopy). Images acquired (.czi format) were analyzed with Zen® software (Zeiss, Germany). Mosaic images were stitched together automatically by the software with a 5% overlap and a maximum shift of 10%. All images were aligned in the same orientation, with the cortical surface at the top and the white matter (WM) at the bottom. For each section, three regions of interest (ROIs) were defined: internal (IT), located in the dorsal part of the SSS; intermediate (IM), located in the dorsal part of the external surface of the anterior part of the posterior ectosylvian gyrus; and external (ET), located ventrally to the intermediate ROI on the anterior part of the posterior ectosylvian gyrus. These ROIs were drawn on relatively straight parts of the cortical surface using Zen® software (width: 1000 μm; height: 1250–2000 μm, depending on the distance between the cortical surface and WM; Figure 2). Cortical layers within each ROI were identified based on cell density and morphology.

Neurons and glial cells were distinguished based on their morphology and laminar location. Glial cells were identified by their small, darkly stained, ovoid nuclei (García‐Cabezas et al., 2016). Pyramidal neurons were recognized by their triangular soma, and a single large apical dendrite that arises from the apex of the cell body (Weedman & Ryugo, 1996). Large pyramidal neurons shared the same morphological characteristics but exhibited a greater diameter and were typically located in layer V (Weedman & Ryugo, 1996). Granular neurons were subclassified based on soma morphology: spindle‐shaped granular neurons exhibited an elongated fusiform soma, while spheroid granular neurons had a round or oval soma (DeFelipe et al., 2013). Cajal–Retzius‐like cells, located in layer I, were identified by their pear‐shaped or ovoid soma, often appearing in a monopolar or multipolar configuration, with radiating dendrites and a characteristic descending axon (Marín‐Padilla, 2015).

The thickness of each layer was measured by one experimenter and total cortical thickness for each ROI was obtained by summing the thicknesses of the six layers. All measurements are presented as mean ± standard deviation, with the thickness of each layer also expressed as a mean percentage of the total cortical thickness. Data from the internal ROI of the posterior section of the right hemisphere of animal 03133 was missing from all analyses due to a cresyl violet staining issue. In addition, four layer thickness measurements that were either more than two times the interquartile range below the first quartile or above the third quartile were considered outliers (*animal 13155: left, anterior section, intermediate ROI, Layer 1; animal 13507: left, medial section, internal ROI, Layer 4; animal 03133: right, posterior section, external ROI, Layer 6; animal 13403: right, medial section, internal ROI, Layer 1*). They were not included in layer thickness means and precluded the calculation of cortical thickness for the corresponding ROIs. For each animal, hemisphere, section, ROI, cortical layer, and cell type, the diameters of five randomly selected cells were measured manually. The mean minimum and maximum diameters were then calculated across animals, hemispheres, sections, and ROIs for each layer and cell type. The percentage of the layer's surface covered by cresyl violet‐stained cells was used to estimate cell density. Coverage of less than 25% was considered low density, 25%–75% moderate density, and more than 75% high density.

#### 2.4.2 PV and MBP immunostaining: Fluorescence acquisition

The 12 PV‐ and the 12 MBP‐immunostained sections selected for further analysis were adjacent to those selected for cresyl violet staining. These sections were imaged using a digital slide scanner Axio Scan.Z1 (ZEISS®, Germany, with a 20× objective, in fluorescence). Mosaic images were automatically stitched together by the Zen® software with a 5% overlap and a maximum shift of 10%. The ROIs and cortical layers defined on cresyl violet‐stained sections were precisely reproduced on the immunostained sections using Zen® software. The acquired images (.czi format) were analyzed with QuPath (Bankhead et al., 2017), an open‐source software for bioimage analysis, allowing automated detection and consistent cell quantifications across sections. All images were aligned in the same orientation, with the cortical surface at the top and the WM at the bottom. PV‐immunoreactive (PV‐ir) cell bodies represent a subpopulation of GABAergic interneurons, characterized by their morphology. Spheroid and spindle‐shaped interneurons were distinguished using the same morphological criteria as for cresyl violet staining. Polygonal cells were identified as multipolar cells with an angular soma (DeFelipe et al., 2013; Graïc et al., 2024). PV‐ir cell bodies within each ROI were automatically counted using QuPath (Images: 16‐bit intensity range; Detection channel: CY3; Requested pixel size: 0.3256 μm; Nucleus parameter: 8 μm background radius – 4 μm median filter radius; Min area: 80 μm^2^; Max area: 600 μm^2^; Cell expansion: 2 μm; Pixel intensity threshold: 950–15,000). All measurements are presented as median cell density in each ROI. Similar to cresyl violet staining, densities of PV‐ir cell bodies, PV‐ir fibers and MBP‐immunoreactive (MBP‐ir) myelin were assessed based on the proportion of a layer's surface covered by the immunostaining (low: <25%, moderate: 25%–75%, or high: >75%). Within each cortical layer PV‐ir fiber length was measured manually and categorized as small (<30 μm), medium (30–160 μm), or long (>160 μm).

## 3 RESULTS

### 3.1 Cresyl violet staining

**FIGURE 3:**
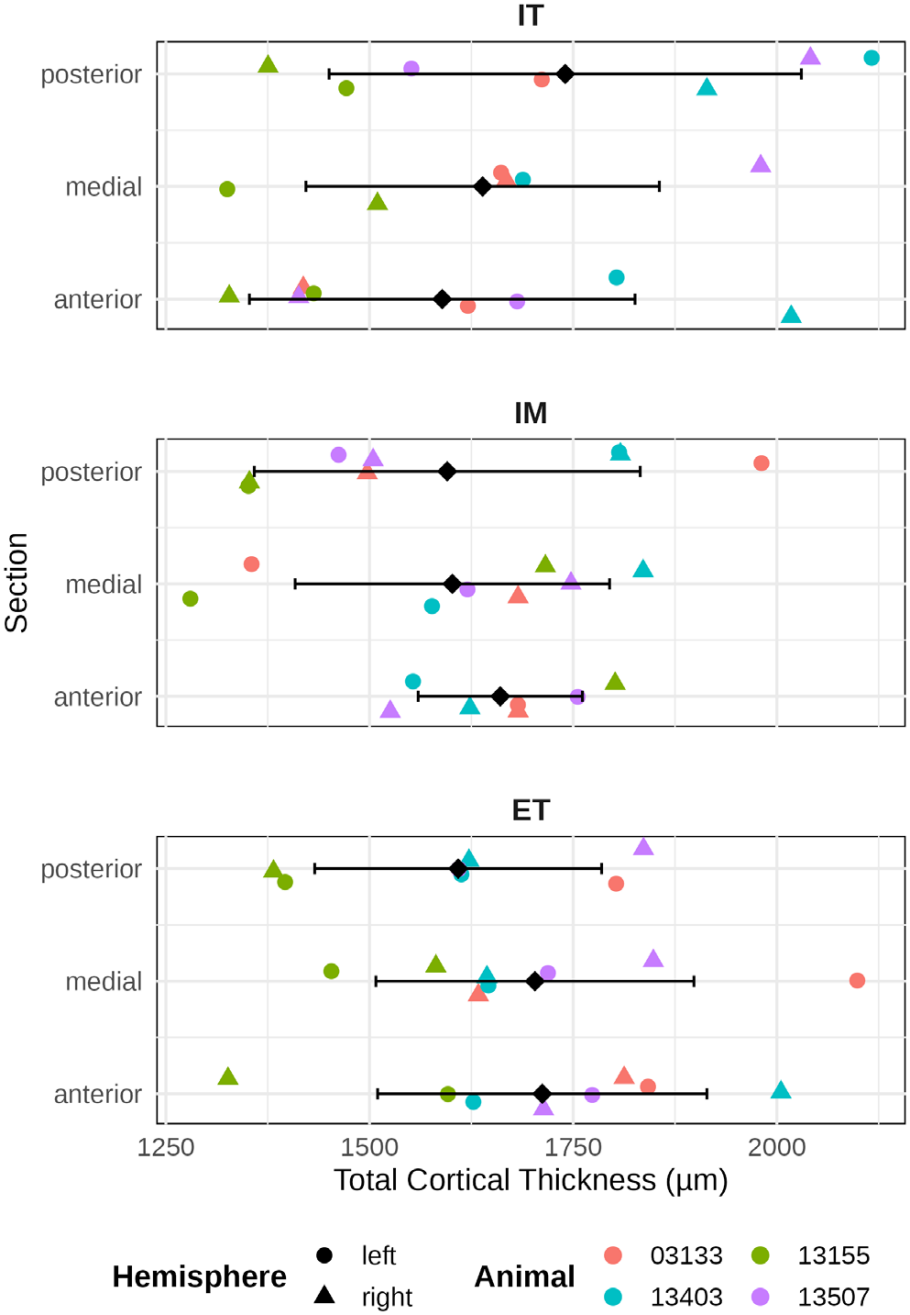
Total cortical thickness (μm) of the sheep auditory cortex. Total cortical thickness was measured within the external (ET), intermediate (IM) and internal (IT) ROIs of the anterior, medial, and posterior sections, for both hemispheres (indicated by point size), in four animals (distinguished by color). The total thickness of the auditory cortex varied between 1280 μm and 2116 μm across ROIs, sections, hemispheres, and animals.

Cresyl violet staining enabled the delineation of the cortical thickness (distance between the cortical surface and the WM) of the ovine auditory cortex (Figure 2). No clear, consistent trends were observed that could account for the variability in total cortical thickness across animal, hemisphere, section, or ROI (Figure 3). The mean total thickness of the auditory cortex was 1649 μm, with a standard deviation of 205 μm.

### 3.2 Layer identification

**FIGURE 4:**
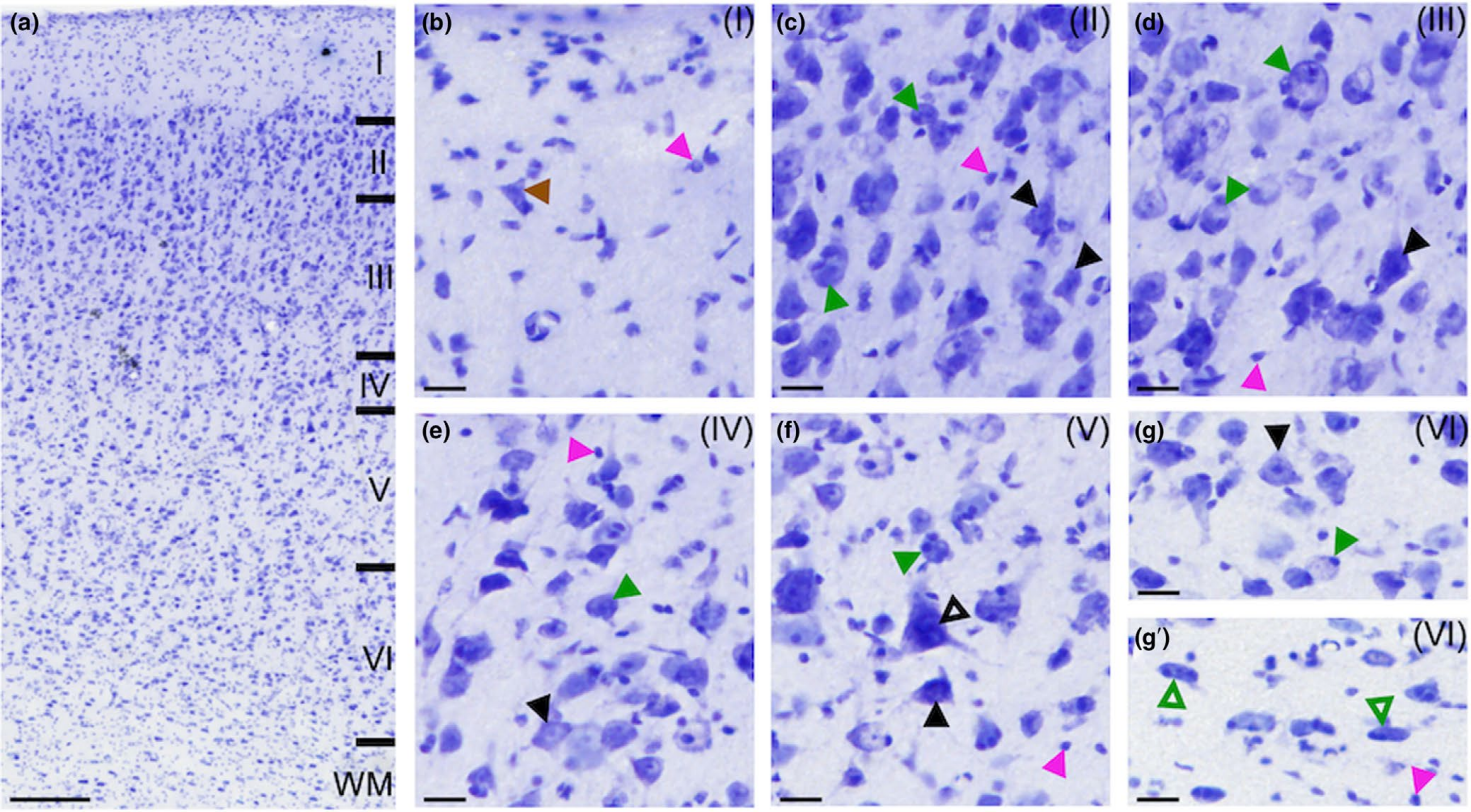
Nissl‐stained section of the sheep auditory cortex. (a) Identification of six cortical layers (I, II, III, IV, V, VI) and the white matter (WM); (b) Glial cells (pink arrowhead) and Cajal–Retzius‐like cells (brown arrowhead) of layer I; Spheroid granular cells (green arrowhead) and pyramidal cells (black arrowhead) of layers II (c), III (d), IV (e), V (f), and VI (g); (f) Large pyramidal cells of layer V (unfilled black arrowhead); (g’) Spindle‐shaped granular cells of layer VI (unfilled green arrowhead). Note that glial cells were present in all six layers (pink arrowheads). Data shown are from the external ROI, of the anterior section, of the left hemisphere, of animal 03133. Scale bars: 200 μm (a); 20 μm (b–g’).

Examination of cellular organization revealed six cortical layers in the sheep auditory cortex. Each layer had distinct variations in cell density and morphology, which were consistent across sections and ROIs (Figure 4, Table 2). In detail, layer I was composed mainly of small, sparsely distributed glial cells, with mean diameters ranging from 6.2 μm ± 1.4 to 11 μm ± 2.5. In addition, a few Cajal–Retzius‐like cells were present, with diameters between 12.1 μm ± 2.2 and 18.7 μm ± 2.9 (Figure 4b). These cells were distributed throughout the layer. The boundary with layer II was easily distinguished by a clear change in cell type and density. Layer II was primarily characterized by spheroid granular cells, with diameters ranging from 13.7 μm ± 2.4 to 20.4 μm ± 2.8. These cells were aggregated in strings throughout the layer. Additionally, a few pyramidal cells with triangular‐shaped cell bodies were observed, with diameters ranging from 13.8 μm ± 2.3 to 21.3 μm ± 3.3 (Figure 4c). Granular and pyramidal cells were evenly distributed across the layer. Moving deeper into the cortex, the cell aggregation decreased, delineating the border between layers II and III. In layer III, spheroid granular cells (diameter: 14.4 μm ± 2.2–19.7 μm ± 2.5) were more numerous than pyramidal cells (diameter: 14.6 μm ± 2.2–21.8 μm ± 3), both were distributed throughout the layer (Figure 4d). Distinguishing between layers III and IV proved challenging. However, a slight decrease in overall cell density could be observed in layer IV compared with layer III. In layer IV, spheroid granular cells (mean diameter: 14.9 μm ± 2.2–21.5 μm ± 3.1) were more numerous than pyramidal cells (mean diameter: 13.3 μm ± 2–19.6 μm ± 3), both distributed throughout the layer (Figure 4e). The upper part of layer V contained a few large pyramidal cells (Figure 4f, mean diameter: 23.2 μm ± 3.8–33.4 μm ± 5.2), which marked the distinction between layers IV and V. Layer V also consisted of smaller pyramidal cells (mean diameter: 14.1 μm ± 2.2–19.6 μm ± 3) and a larger number of spheroid granular cells (mean diameter: 14.5 μm ± 2 to 20.8 μm ± 2.9), both distributed throughout the layer (Figure 4f). A slight increase in overall cell density could be observed in layer VI compared to layer V. Layer VI contained both spheroid granular cells, with mean diameters ranging from 14.5 μm ± 2.3 to 19.8 μm ± 2.7, and pyramidal cells, with mean diameters from 13.9 μm ± 2.2 to 20.7 μm ± 3.3, both distributed throughout the layer (Figure 4g). Additionally, this layer included spindle‐shaped granular cells arranged parallel to the interface with the WM, aligned along their long axis (Figure 4g’).

**TABLE 2:** Cortical organization of the sheep auditory cortex.

Layer	Cresyl violet staining						Myelin basic protein immunostaining				Parvalbumin immunostaining			
	Mean thickness		Cell		Mean cell diameter		Fiber		Cell		Mean cell diameter		Fiber	
	μm	%	Type	Density	Min (μm)	Max (μm)	Density	Orientation	Density	Type	Min (μm)	Max (μm)	Density	Orientation
I	282 ± 71	17.1 ± 4.3	Glial	Moderate	6.2 ± 1.4	11 ± 2.5	Low	Parallel	Ø	Ø	Ø	Ø	Low	Perpendicular
			Cajal‐like	Low	12.1 ± 2.2	18.8 ± 3								Parallel
II	131 ± 30	7.9 ± 1.8	Granular	High	13.6 ± 2.6	20.4 ± 3.5	Low	Perpendicular	Low	Spheroïd	15.4 ± 3	19.2 ± 3.7	Moderate	Perpendicular
										Polygonal	18 ± 5.6	22 ± 4.5		
			Pyramidal	Low	13.7 ± 2.6	21.3 ± 3.3				Spindle‐shaped	22.3 ± 2	22.8 ± 2.4		Parallel
III	342 ± 9	20.7 ± 4.8	Granular	Moderate	14.9 ± 2.2	21.5 ± 3.1	Moderate/High	Perpendicular	High	Spheroïd	14.8 ± 1.8	18.9 ± 2	High	Perpendicular
			Pyramidal	Moderate	14.6 ± 2.2	21.8 ± 3				Polygonal	18 ± 2.2	24.5 ± 3.6		Parallel
										Spindle‐shaped	21.9 ± 3.3	24 ± 2.8		
IV	181 ± 45	10.9 ± 2.7	Granular	Moderate	14.4 ± 2.2	19.7 ± 2.5	Moderate/High	Perpendicular	Moderate	Spheroïd	14.5 ± 2.5	17.4 ± 2.6	High	Perpendicular
			Pyramidal	Moderate	13.3 ± 2	19.6 ± 3				Polygonal	17.8 ± 2.7	25 ± 4.1		Parallel
										Spindle‐shaped	20.4 ± 2.9	23.1 ± 4.1		
V	341 ± 88	20.6 ± 5.3	Granular	Moderate	14.5 ± 2	20.8 ± 2.9	High	Perpendicular	High	Spheroïd	15 ± 1.9	19.2 ± 3	High	Perpendicular
			Pyramidal	Moderate	14.1 ± 2.2	19.6 ± 3				Polygonal	20.2 ± 3.1	27.7 ± 3.8		Parallel
			Large Pyramidal	Low	23.2 ± 3.8	33.4 ± 5.2				Spindle‐shaped	20.5 ± 3.2	24.1 ± 3.7		
VI	376 ± 136	22.8 ± 8.2	Granular	Moderate	14.5 ± 2.3	19.8 ± 2.7	Moderate	Perpendicular	Moderate	Spheroïd	16.6 ± 3.5	20.4 ± 3	Moderate	Perpendicular
			Pyramidal	Moderate	13.9 ± 2.2	20.7 ± 3.3				Polygonal	21.1 ± 3.8	26.1 ± 4.5		Parallel
										Spindle‐shaped	23.1 ± 3.2	25.2 ± 3.3		

**FIGURE 5:**
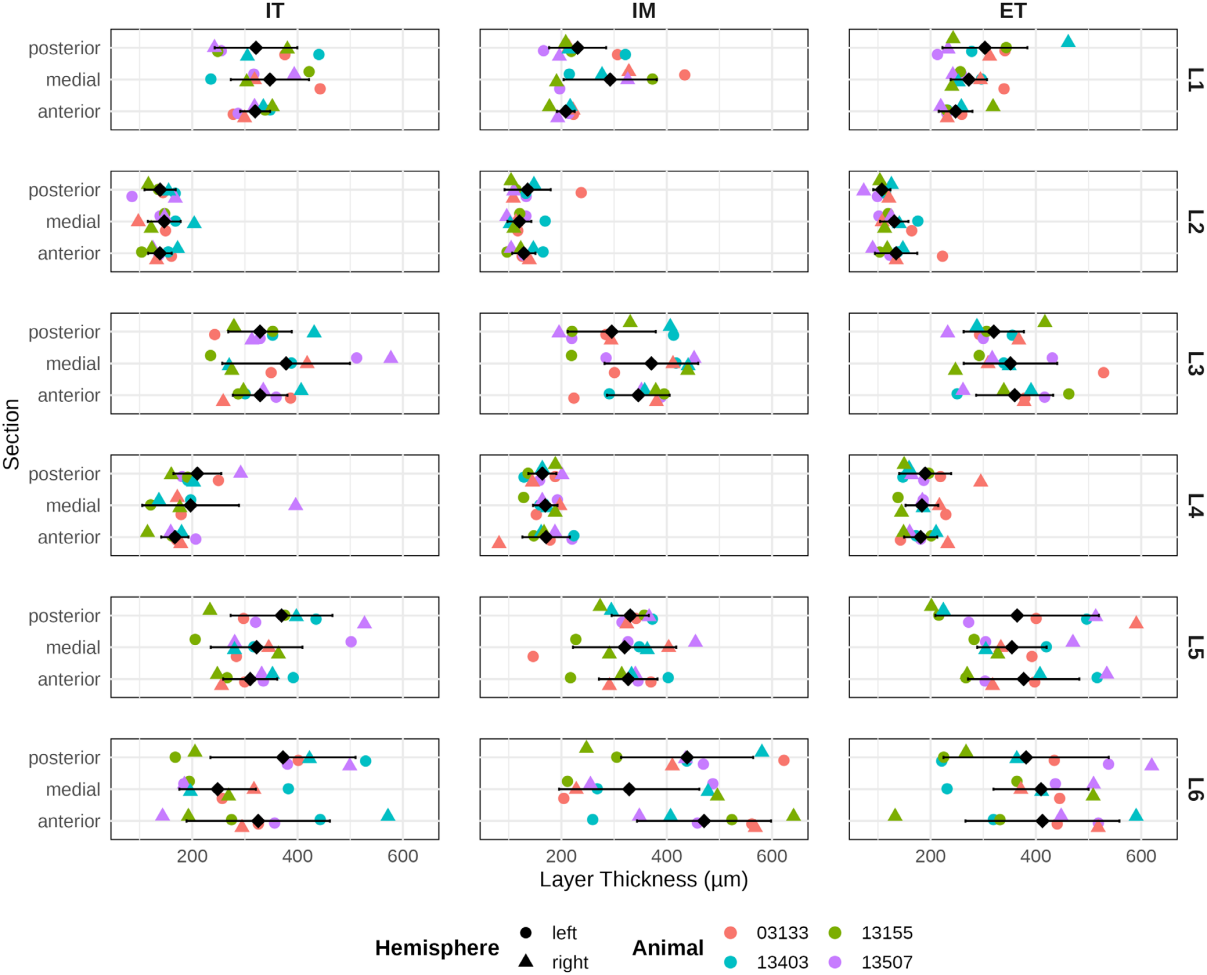
Thickness (μm) of the six cortical layers in the sheep auditory cortex. Thickness of each cortical layer was measured for the anterior, medial, and posterior sections within the internal (IT), intermediate (IM), and external (ET) ROIs. Measurements were performed for both hemispheres (indicated by different shapes), in four animals (distinguished by color). No clear, consistent trends in layer thickness were observed across either animal, hemisphere, section, or ROI.

Based on this cellular organization, the thickness of each of the six cortical layers across sections, for each ROI and hemisphere of the four animals was measured (Figure 5). Layers II and IV were the thinnest (131 μm ± 30; 181 μm ± 45, respectively) and exhibited the lowest variability. These two layers represented 7.9% and 10.9% of the total thickness, respectively. Layer I was thicker (282 μm ± 71) and showed slightly more variability than layers II and IV. The mean thicknesses of layers III and V were similar (342 μm ± 79; 341 μm ± 88, respectively) while layer VI was the thickest (376 μm ± 136) of all layers. The variability observed in the thicknesses of layers I, III, V, and VI did not appear to be linked to any specific criterion, such as ROIs, sections, hemispheres, or animals. Overall, the mean percentage of total cortical thickness of layers I, III, V, and VI was similar, ranging from 17.1% to 22.8% (Table 2).

### 3.3 MBP immunostaining

**FIGURE 6:**
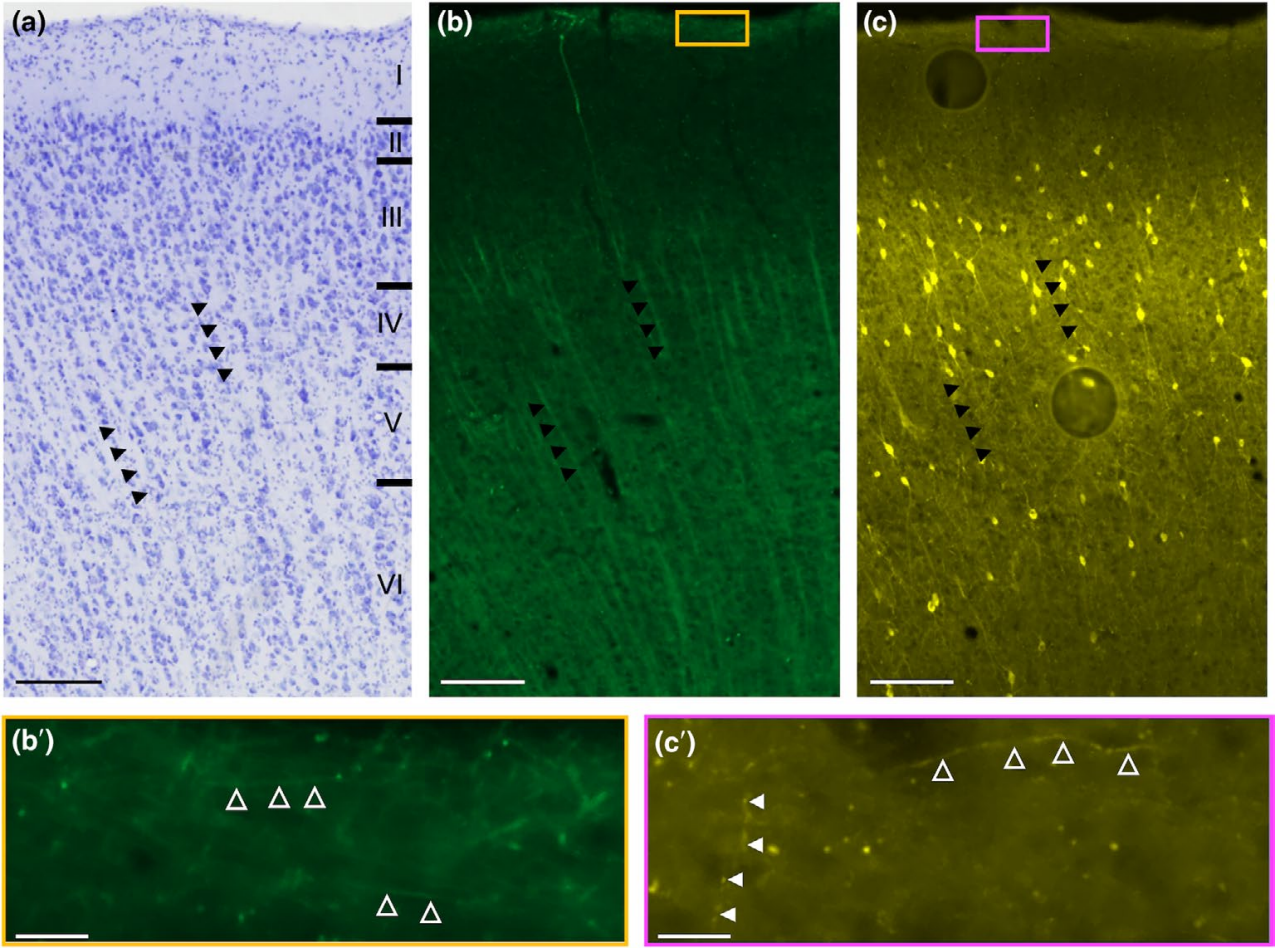
Orientation of fibers and cells in the sheep auditory cortex. (a) Cresyl violet staining revealed cells aligned perpendicular to the cortical surface; (b) MBP staining highlighted fibers with a similar perpendicular orientation (black arrowheads) and parallel fibers in the upper part of layer I (unfilled white arrowhead in b’); (c) PV staining revealed fewer perpendicular fibers and cell bodies; (c’) In the upper part of layer I, PV‐ir fibers were parallel (unfilled white arrowhead) and perpendicular (filled white arrowhead) to the cortical surface. Data shown are from the external ROI, of the posterior section, of the left hemisphere, of animal 13507. Black arrowheads in a, b & c indicate equivalent locations and were defined based on the fibers in b. Scale bars: 200 μm (a–c); 20 μm (b’ and c’).

In the sheep auditory cortex, MBP‐ir myelin was observed for all animals, hemispheres, sections, and ROIs. MBP‐ir myelin was present in all six cortical layers (Figure 6). MBP immunostaining revealed fibers oriented parallel to the cortical surface in the upper part of layer I (Figure 6b’), whereas fibers oriented perpendicular to the cortical surface were observed traversing layers II–VI (Figure 6b).

**FIGURE 7:**
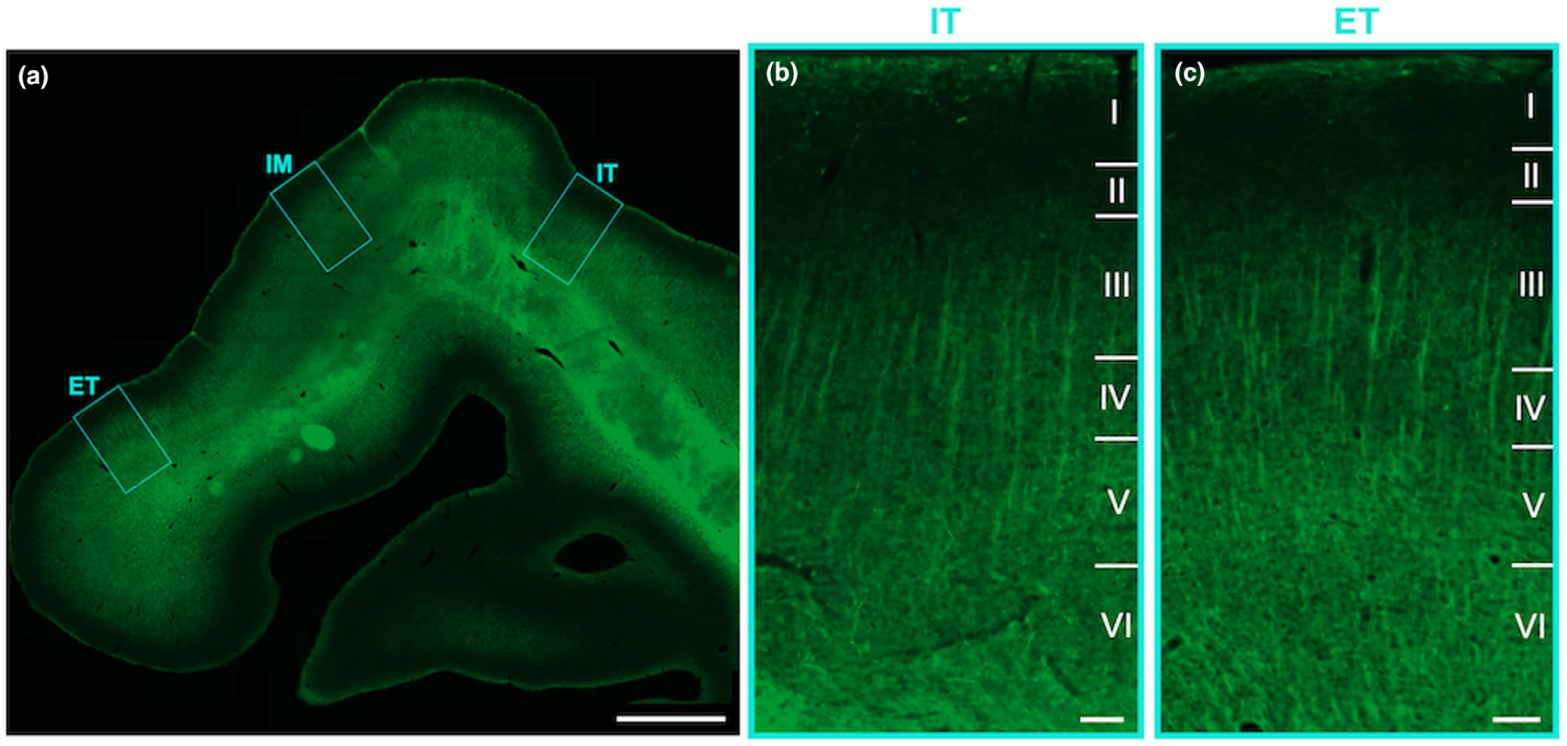
Distribution of MBP‐ir myelin in the sheep auditory cortex. (a) MBP staining in the anterior section of the left hemisphere of animal 13507; (b) Internal (IT) ROI: Low density of MBP‐ir myelin in layers I and II, moderate density in layer VI, and high density in layers III–V; (c) External (ET) ROI: Low density of MBP‐ir myelin in layers I–II, moderate density in layers IV and VI, and high density in layers III and V. Cortical layers, defined using cresyl violet‐stained sections, were precisely reproduced onto the immunostained sections. Scale bars: 2000 μm (a); 200 μm (b and c).

No clear differences in MBP immunostaining across hemispheres, sections, and ROIs were observed for layers I, II, V, and VI. Layers I and II consistently contained a low density of MBP‐ir myelin, while layer VI exhibited a moderate density, and layer V a high density. In contrast, layers III and IV showed greater variability in their MBP‐ir density. Layer III contained either a moderate or high density of MBP‐ir but with a notable prevalence of high density (Figure 7a). Layer IV contained moderate or high MBP‐ir myelin density, with moderate density mainly in external ROIs (Figure 7b), high density predominantly in internal ROIs, while intermediate ROIs had an equal distribution of moderate or high MBP‐ir myelin density.

### 3.4 PV immunostaining

**FIGURE 8:**
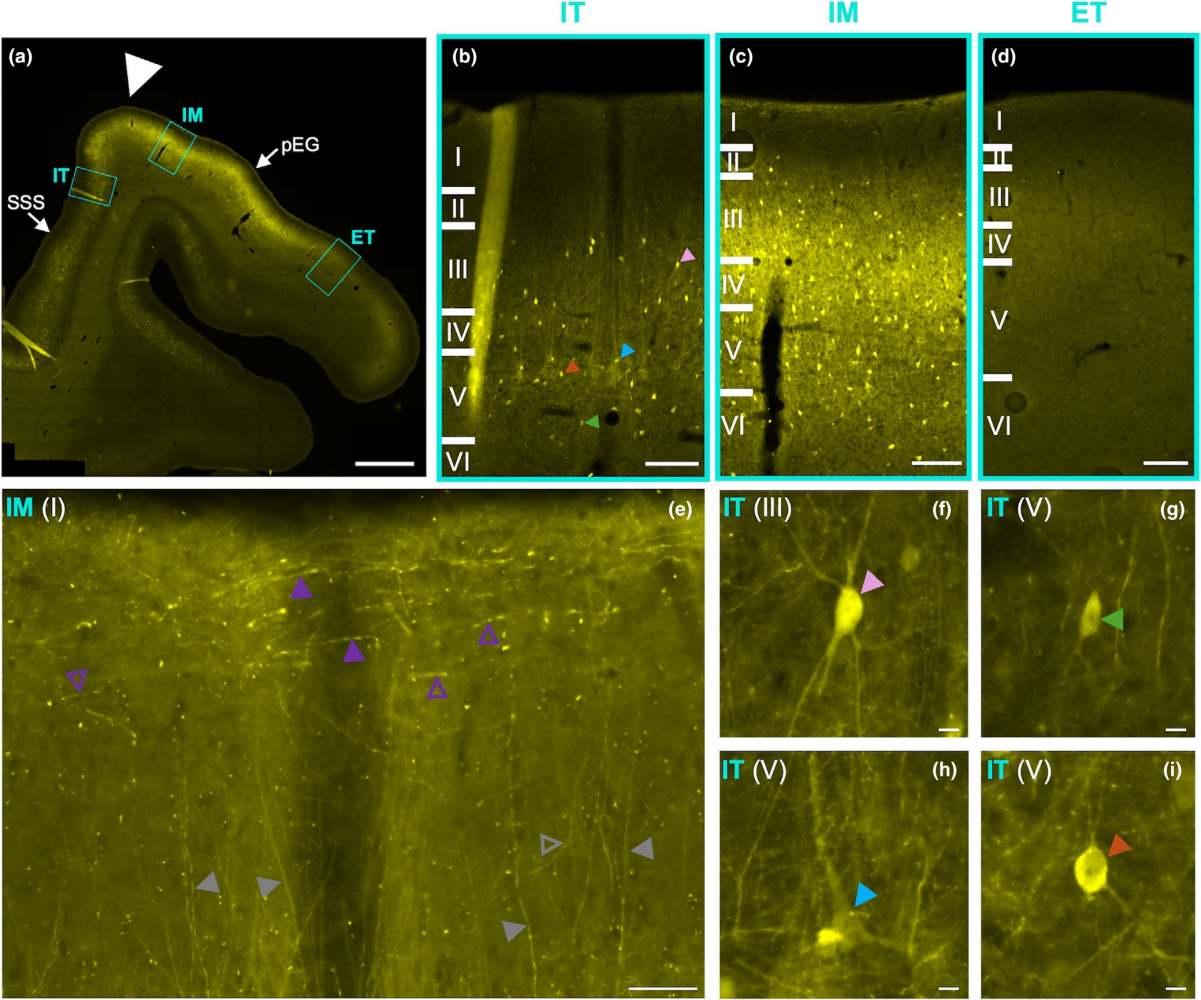
Distribution of PV‐ir cell bodies and PV‐ir myelin in the sheep auditory cortex. (a) The dorsoventral gradient of PV staining in the anterior section of the right hemisphere of animal 13507, with the three ROIs (IT: Internal, IM: Intermediate, ET: External) indicated in blue. The highest density of PV staining was observed in the dorsal part of the posterior ectosylvian gyrus (white arrowhead). The gradient decreased abruptly into the suprasylvian sulcus (SSS) and more gradually along the outer cortical surface (pEG). (b–d) Distribution of PV staining in the three ROIs (IT‐IM‐ET). (e) PV‐ir fiber orientation and length in layer I of the intermediate (IM) ROI, revealing long (filled gray arrowhead) and medium (unfilled gray arrowhead) perpendicular fibers, medium (filled purple arrowhead) and short (unfilled purple arrowhead) parallel fibers. (f–i) GABAergic PV‐ir cell bodies; (f) polygonal interneuron with axonal projections in layer III of the internal (IT) ROI (pink arrowhead); (g) spindle‐shaped interneuron in layer V of the internal (IT) ROI (green arrowhead); (h) basket‐like structure in layer V of the internal (IT) ROI (blue arrowhead) and (i) spheroid interneuron in layer V of the internal (IT) ROI (orange arrowhead). The highest density of PV was observed in the intermediate (IM) ROI, followed by the internal (IT) ROI, and no PV‐ir cell bodies in the external (ET) ROI. Scale bars: 2000 μm (a); 200 μm (b–d); 50 μm (e); 10 μm (f–i).

PV‐ir immunostaining revealed fibers and cell bodies distributed throughout the sheep auditory cortex. In general, a dorsoventral gradient of staining was observed, with the highest density being observed in the dorsal part of the posterior ectosylvian gyrus between the internal and intermediate ROIs (Figures 8 and S1). The dorsoventral gradient decreased abruptly into the SSS, with a nearly complete absence of cell body staining after the internal ROI. On the outer cortical surface of the posterior ectosylvian gyrus, the gradient decreased more gradually, with the external ROI exhibiting an almost complete absence of cell body staining (Figure 8d). However, short PV‐ir fibers were still observed in this ROI despite the lack of stained cell bodies.

In the majority of the internal and intermediate ROIs, medium to long fibers were observed in layers I and II, whereas layers III, IV, V, and VI contained predominantly long fibers (Figure 8e). The highest density of PV‐ir fibers was observed in layers III, IV, and V, followed by layers II and VI, and the lowest in layer I. Across all six cortical layers, fibers oriented both perpendicular and parallel to the cortical surface were observed, with more perpendicular (Figure 8e). Fibers with diverse orientations were also observed in the upper part of layer I (Figure 6c’). In addition, large basket‐like structures were observed around unstained cell bodies in the upper part of layer V, with a mean diameter ranging from 34.8 μm ± 8.7 to 42.9 μm ± 8.3 (Figure 8h).

PV‐ir cell bodies were observed in layers II, III, IV, V, and VI, but not in layer I (Figure 8b,c). They consisted of subpopulations of GABAergic interneuron cell bodies, including spheroid, polygonal, and spindle‐shaped (Table 2, Figure 8f–i). The density of PV‐ir cell bodies varied across sections and ROIs. The highest median density was found in the intermediate ROI (65.9 cells/mm^2^), followed by the internal ROI (50.2 cells/mm^2^), and the lowest density in the external ROI (17.2 cells/mm^2^, Figure 8b–d).

The density of PV‐ir cell bodies varied by layer, with the highest density in layers III and V, followed by a moderate density in layers IV and VI, a low density in layer II, and no interneurons in layer I (Figure 8c). In detail, layer II consisted of a sparse distribution of spheroid interneurons, along with rare polygonal and spindle‐shaped interneurons. In layers III and IV, polygonal interneurons, with axonal projections, and spheroid interneurons were present. Spindle‐shaped interneurons were also occasionally observed in these layers. In layers V and VI, polygonal interneurons were present, along with a smaller number of spheroid interneurons and rare spindle‐shaped interneurons. The minimum and maximum mean diameter for each cell type is presented in Table 2.

## 4 DISCUSSION

In this study, we provided a detailed description of the cytoarchitecture and myeloarchitecture of the sheep auditory cortex, shedding light on its structural organization. Our analysis was guided by the localization reported by Michaloudi et al. (1986). However, it should be noted that due to methodological limitations of that study (number and location of the injection sites), the auditory cortex likely extends beyond the cortical regions examined here. Therefore, it is a limitation of the current study that we cannot be sure whether our analysis covered the full extent of the auditory cortex or not.

The mean cortical thickness of the sheep auditory cortex (1649 μm; min: 1280 μm; max: 2116 μm) was consistent with the cortical thickness of other regions of the sheep brain reported in previous studies. Among these regions, the primary visual cortex has been reported to be the thinnest (1488 μm; Graïc et al., 2022), followed by the auditory cortex and the orbitofrontal cortex (1697 μm; Gerussi et al., 2022). In contrast, the motor cortex exhibits the greatest thickness (1838 μm; Peruffo et al., 2019). This pattern of cortical thickness across regions is broadly consistent with findings in other mammalian species (Gordon et al., 2024; Palomero‐Gallagher & Zilles, 2019; Wagstyl et al., 2015). Moreover, the six‐layer laminar organization observed in the sheep auditory cortex is consistent with findings in the auditory cortices of other mammals (reviewed in Kaas, 2011). This suggests a somewhat conserved structural organization, and potentially common auditory processing mechanisms, across mammalian species.

In the cat, the primary auditory cortex is situated in the middle of the ectosylvian gyrus (Winer, 1984). It is characterized by small, round, densely packed somata, and small pyramidal neurons in layer II, bitufted, pyramidal and multipolar cells, in a layer III that is often divided into two parts (IIIa and IIIb), containing smaller and larger pyramidal cells, respectively. Layer IV is usually relatively narrow where round and multipolar cells dominate. Layer V is dominated by larger pyramidal cells, with a layer Va characterized by fibers (neuropil) and a more cell‐rich Vb. Layer VI is traversed by WM fascicles and is composed of large pyramidal and fusiform cells oriented mostly horizontally. Layers III, IV, and V are cytochrome oxidase positive, express parvalbumin and acetylcholine esterase more than in non‐primary areas (Wallace et al., 1991).

In dogs, the primary auditory cortex was also found in the anterior and middle ectosylvian gyrus (Tunturi, 1962), and in a posterior pseudosylvian field in ferrets (Kaas, 2011). Malinowska and Kosmal (2003) described the dog ectosylvian gyrus, and the primary auditory cortex homologue located in its anterior part. It is characterized by similar features as that of the cat, namely, a deeper cortex compared with bordering areas, owing to larger supragranular layers (I–III). Layer IV in particular was found to contain multiform and small pyramidal cells. In the middle part of the ectosylvian gyrus, layer IV was progressively invaded by cells from layers III and V. Similar patterns were found in the ferret (Bajo et al., 2007) and in primates (Hackett et al., 2001).

Among the six cortical layers of the sheep auditory cortex, layer II was the thinnest, which is consistent with other regions of the sheep brain (Graïc et al., 2022; Rose, 1942) and in the cortex of other cetartiodactyls (Graïc et al., 2024; Hof et al., 1999). In addition, this layer exhibited the highest cell density, consistent with findings in mammals (Winer, 1985). Layer IV was also relatively thin but its boundaries were more challenging to delineate from adjacent layers. These observations mirror findings in other primary sensory areas, such as the primary visual cortex of sheep (Graïc et al., 2022; Rose, 1942), where layer IV is present but difficult to identify.

Immunostaining for MBP and PV revealed the highest fiber density in layers III–V, which is consistent with studies in other mammals, where these layers are known to harbor dense projections (Hof et al., 1999; Jones et al., 1995; Kaas, 2011; Wallace et al., 1991). The dense MBP‐ir myelin and PV‐ir fibers suggest a developed network of connections mediated by interneurons, facilitating both local processing, cortico‐cortical, and thalamo‐cortical communication within the sheep auditory cortex.

Interestingly, we identified basket‐like structures exclusively in the upper part of layer V using PV immunostaining. These structures, based on their distinctive morphology and size, likely surround large pyramidal cells. This could be confirmed by combining PV immunostaining with SMI‐32 staining (del Río & DeFelipe, 1994), which preferentially labels pyramidal neurons in layers III and V (Berger et al., 2017; Campbell & Morrison, 1989). Such large pyramidal cells were observed in the upper part of layer V with cresyl violet staining. This arrangement suggests that the basket‐like structures, known to be PV‐immunoreactive (DeFelipe et al., 2013), may modulate the activity of pyramidal cells through GABAergic synapsing, potentially playing a pivotal role in regulating excitatory output from layer V, which was previously reported in other mammals (Hof et al., 1999). Additionally, long axonal projections were observed extending into layer II, which may correspond to the projections of large pyramidal neurons. This anatomical configuration highlights a complex interplay between inhibitory and excitatory elements, with basket‐like structures contributing to local inhibitory regulation of excitatory pyramidal cells, crucial for auditory information processing (Studer & Barkat, 2022).

PV staining has already been used as an indicator of the location of the primary auditory cortex (Kaas & Hackett, 2000). In our study, a high density of PV‐ir cell bodies was observed in the dorsal part of the posterior ectosylvian gyrus, with staining decreasing ventrally, both into the SSS and along the outer cortical surface. These observations suggest that the sheep *primary* auditory cortex is located in the dorsal posterior ectosylvian gyrus, maybe between the internal and intermediate ROIs of the anterior and medial sections, which broadly corresponds to neural tracing results (Michaloudi et al., 1986). While this evidence provides a strong anatomical basis for identifying the *primary* auditory cortex, functional studies (e.g., electrophysiology and/or fMRI) would help in precisely mapping its spatial boundaries and potential tonotopic organization.

## 5 CONCLUSION

In this study, we delineated six cortical layers within the sheep auditory cortex in the anterior part of the posterior ectosylvian gyrus, with distinct cell types, distribution, and fiber organization. Additionally, we observed increased parvalbumin staining in the dorsal part of this region, which may correspond to the primary auditory cortex. These findings are consistent with existing literature on the auditory cortex in sheep and other mammalian species. Together, these results suggest that the organization of the auditory cortex is conserved across species, supporting the idea of an evolutionary conservation of auditory processing mechanisms in mammals.

## AUTHOR CONTRIBUTIONS

Camille Pluchot: conceptualization, data curation, formal analysis, investigation, methodology, validation, visualization, writing – original draft preparation, writing – review and editing; Melody Morisse: conceptualization, data curation, formal analysis, investigation, methodology, validation, visualization, writing – review and editing; Maryse Meurisse: conceptualization, data curation, formal analysis, investigation, methodology, validation, visualization, writing – review and editing; Jean‐Marie Graïc: methodology, validation, writing ‐ review and editing; Elodie Chaillou: conceptualization, formal analysis, funding acquisition, methodology, project administration, validation, writing – review and editing; Scott Love: conceptualization, data curation, formal analysis, funding acquisition, investigation, methodology, project administration, validation, visualization, writing – original draft preparation, writing – review and editing.

## Supporting information


**Data S1:** Supporting Information.

## Data Availability

The data generated during the current study are available in the associated Zenodo repository: 10.5281/zenodo.14824250. The repository includes compressed (quality 70%) versions of the scanned sections with their annotations (.czi file format) and spreadsheets containing layer thickness and cell diameter measurements. Compressed files are being made available, rather than uncompressed, to comply with the size limit of a Zenodo repository. The quality of the compressed images is more than sufficient to replicate the current results; however, uncompressed versions can be obtained from the authors.
