# Supplementary material for: Cytoarchitecture and myeloarchitecture of the sheep auditory cortex
Source: J Anat. 2025 Nov 12;249(2):233–46. doi: 10.1111/joa.70072 (PMC13339931; doi:10.1111/joa.70072)
Supplement: Supplementary file 1 — Data S1: Supporting Information. [file JOA-249-233-s001.docx]

**Supplementary material**


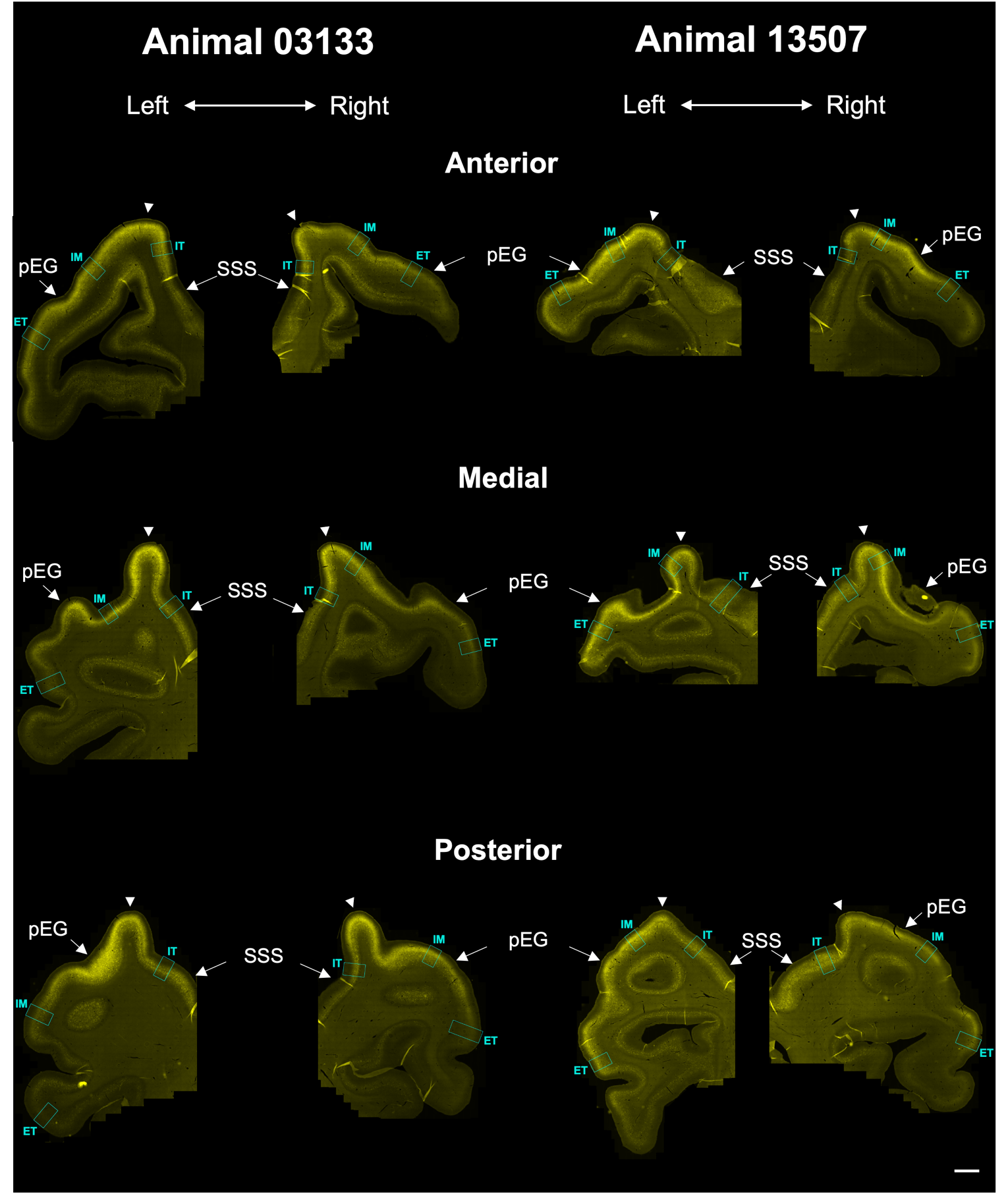


**Supplementary Figure 1. Dorsoventral gradient of PV staining in the sheep auditory cortex.** *The three sections (anterior, medial, posterior) are represented for both hemispheres of animals 03133 and 13507. The internal (IT), intermediate (IM) and external (ET) ROIs are indicated in blue for each section.* *A dorsoventral gradient in PV staining density is observed, with the highest density in the dorsal part of the posterior ectosylvian gyrus (pEG, white arrowhead). The gradient decreases abruptly into the suprasylvian sulcus (SSS) and more gradually along the outer cortical surface (pEG). Scale bar at bottom right applied for all sections: 2000 µm.*
